# Risk Factors and Incidence of Hepatocellular Carcinoma in Southeast Iran

**DOI:** 10.5812/kowsar.1735143X.710

**Published:** 2011-08-01

**Authors:** Seyed Yasser Saiedi Hosseini

**Affiliations:** 1 Baqiyatallah Research Center for Gastroenterology and Liver Diseases, Baqiayatallah University of Medical Sciences, Tehran, IR Iran

**Keywords:** Hepatocellular carcinoma, Risk factors

Dear Editor,

Hepatocellular carcinoma (HCC) is the fifth most common malignancy in the world and is estimated to cause approximately half a million deaths annually [1]. The incidence of HCC varies widely according to geographic location. The distribution of HCC also differs among ethnic groups and regions within the same country; the extreme difference in distribution of HCC isprobably due to regional variations in exposure to different risk factor such as hepatitis viruses and environmental pathogens [2]. A variety of important risk factors for HCC development has been identified. It includes the hepatitis B carrier state, chronic hepatitis C virus (HCV) infection, hereditary hemochromatosis, and cirrhosis of almost any cause [3]. I read valuable article of Darvish Moghaddam et al. [4] about incidence of HCC in southeast of Iran; it seems we need more discussion about risk factors in this region, particularly in Kerman province. They also said "It seems that the incidence of HCC is much higher in Kerman compared to other parts of Iran and some portion of the lower HCC incidence rate in Iran-as a whole than in Kerman-could be due to the low accuracy of the national cancer registry compared to the sensitivity of our multiple sources of active case findings in Kerman" but it seems that two major risk factor for HCC were forgotten and maybe the higher incidence of HCC in Kerman is due to higher prevalence of HBV and HCV infections not just for greater exposure to dietary aflatoxin in the past. Our available data on HBV and HCV infections in Iran [5][6] shows that we have no data about these two major risk factors for HCC in Kerman province. In this way we can say we need more studies on HBV , HCV and other chronic liver diseases as a risk factor of HCC in Kerman province that could be helpful in public health management too.
